# Binding mode and free energy prediction of fisetin/β-cyclodextrin inclusion complexes

**DOI:** 10.3762/bjoc.10.296

**Published:** 2014-11-27

**Authors:** Bodee Nutho, Wasinee Khuntawee, Chompoonut Rungnim, Piamsook Pongsawasdi, Peter Wolschann, Alfred Karpfen, Nawee Kungwan, Thanyada Rungrotmongkol

**Affiliations:** 1 Department of Biochemistry, Faculty of Science, Chulalongkorn University, Bangkok 10330, Thailand; 2 Nanoscience and Technology Program, Graduate School, Chulalongkorn University, Bangkok, 10330, Thailand; 3 National Nanotechnology Center (NANOTEC), National Science and Technology Development Agency (NSTDA), 111 Thailand Science Park, Thanon Phahonyothin Tambon Khlong Nueng, Amphoe Khlong Luang, Pathum Thani 12120, Thailand; 4 Department of Pharmaceutical Technology and Biopharmaceutics, University of Vienna, Vienna 1090, Austria; 5 Institute of Theoretical Chemistry, University of Vienna, Vienna 1090, Austria; 6 Department of Chemistry, Faculty of Science, Chiang Mai University, Chiang Mai 50200, Thailand

**Keywords:** cyclodextrin, fisetin, flavonoid, MM-PBSA, molecular dynamics simulation, QM-PBSA

## Abstract

In the present study, our aim is to investigate the preferential binding mode and encapsulation of the flavonoid fisetin in the nano-pore of β-cyclodextrin (β-CD) at the molecular level using various theoretical approaches: molecular docking, molecular dynamics (MD) simulations and binding free energy calculations. The molecular docking suggested four possible fisetin orientations in the cavity through its chromone or phenyl ring with two different geometries of fisetin due to the rotatable bond between the two rings. From the multiple MD results, the phenyl ring of fisetin favours its inclusion into the β-CD cavity, whilst less binding or even unbinding preference was observed in the complexes where the larger chromone ring is located in the cavity. All MM- and QM-PBSA/GBSA free energy predictions supported the more stable fisetin/β-CD complex of the bound phenyl ring. Van der Waals interaction is the key force in forming the complexes. In addition, the quantum mechanics calculations with M06-2X/6-31G(d,p) clearly showed that both solvation effect and BSSE correction cannot be neglected for the energy determination of the chosen system.

## Introduction

**Figure 1:**
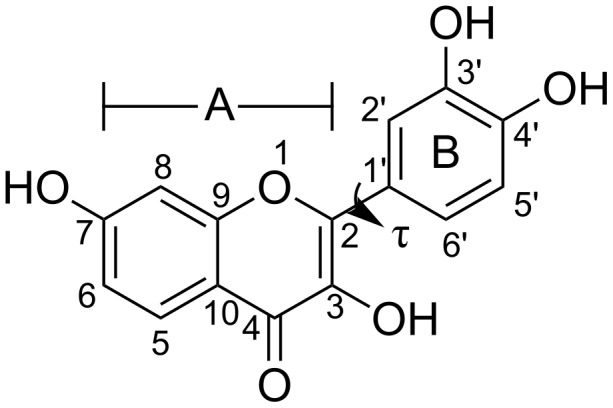
Chemical structure of fisetin with the definition of the A- and B-rings (chromone and phenyl subunits). The atomic labels and the torsional angle (τ) between both aromatic rings are given.

Flavonoids are polyphenolic compounds which are found in many plants as well as in several microorganisms [1–2]. They are herbal secondary metabolites with a wide range of biological and pharmacological activities and are used as therapeutic drugs having many benefits for protection and medical treatment because of their high potency [3–4]. Fisetin (3,3',4',7-tetrahydroxyflavone, 2-(3,4-dihydroxyphenyl)-3,7-dihydroxy-chromen-4-one, Figure 1), one flavonoid in the subclass of flavonols, is found in smoke tree (*Cotinus coggyria*) [5]. It is also present in many fruits and vegetables such as strawberries, grapes, apples, lotus roots, cucumbers and onions [6]. Fisetin has many interesting biological activities and particularly pharmacological properties, including antioxidant, anti-inflammatory, anticarcinogenic and antiviral activities [7]. It was found that fisetin can prevent oxidation which may lead to neuronal cell death [8], and it stimulates cell division of neural cells through extracellular signal-regulated kinase (Erk) activity [9]. This process increases the ability of long-term memory and the efficiency of memory in mice [10]. Fisetin has also been found to induce apoptosis of carcinoma cells via caspase 3 cascade activation [11–12], to inhibit proliferation of human colon (HT-29) cancer cells [13], and to protect against the carcinogen benzo[*a*]pyrene activated lung cancer [14]. In addition, fisetin can stimulate Nrf2 and HO-1 gene expressions that are important in mechanisms of cell defense and cell protection from oxidative conditions [15]. Though several pharmaceutical uses of fisetin are possible, the application is frequently confined by its low water solubility (<1 mg/g) [16].

**Figure 2:**
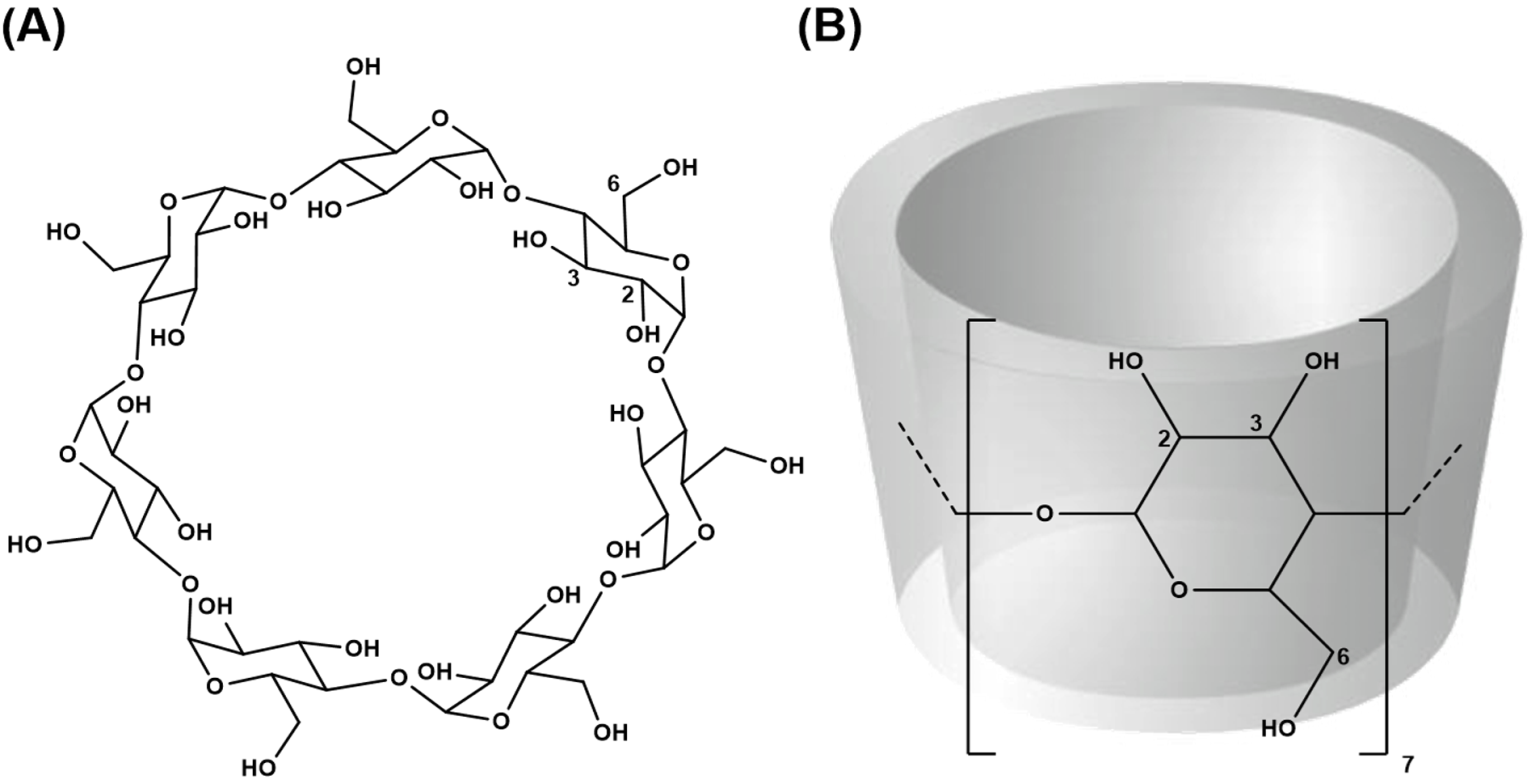
(A) Chemical structure of β-CD and (B) its truncated cone shape.

β-Cyclodextrin (β-CD, Figure 2) is a cyclic oligosaccharide composed of seven α-D-glucopyranose units linked by the α-1→4 glycosidic bonds. The shape of β-CD is that of a truncated cone with hydroxy groups orientated at the rims of the cavity. Its hydroxy groups are divided into two types: the primary hydroxy groups at C6 and the secondary hydroxy groups at C2 as well as C3. At position C6, the primary hydroxy groups of the glucose residues are arranged at the narrow rim, whilst the secondary hydroxy groups are located at the wider rim of the truncated cone. This structural characteristic of β-CD leads to the formation of a relatively hydrophobic cavity [17–19]. In pharmaceutical applications, β-CD has been mostly used as a drug carrier, stabilizer and additive by the formation of host–guest complexes with increased solubility and consequently better bioavailability of low water soluble organic compounds (i.e*.,* drugs) [20–23]. The inclusion complex can also improve ligand stability against exposure to strong UV light and high temperatures [24–25].

In recent years, computational approaches have played a significant role in monitoring inclusion complexation between cyclodextrin and guest molecules [26–27] at the molecular level [28–29]. Molecular dynamics (MD) simulations were used to describe the molecular mechanisms of inclusion complexation between the flavonoids quercetin/myricetin and cyclodextrin in comparison with the experimental results from ^1^H NMR spectroscopy [30]. Choi and coworkers [31] also reported a theoretical study based on MD simulations in order to understand the two flavonoids/β-CD complexes, hesperetin and naringenin complexes, in aqueous solution. The PM3 method was applied to calculate the energy regarding the antioxidant property of the flavonoid chysin in the complex with β-CD [32]. Interestingly, the molecular docking study on the fisetin/β-CD complex [33] suggested that the chromone ring (A-ring defined in Figure 1) of fisetin inserted into the β-CD hydrophobic cavity leads to a more stable complex than the insertion of the phenyl ring (B-ring). This is in contrast to the QM studies based on the SAM1, B3LYP and MPW1PW91 methods [28]. Since in these two previous studies, the fisetin/β-CD inclusion complex was optimized in gas phase, the A- or B-rings of fisetin were not well inserted, but only partially occupied in the cavity, while the other ring entirely stayed outside of the β-CD moiety. Herein, the host–guest inclusion complexation between fisetin and β-CD in aqueous solution was investigated by the multiple MD simulations with three different initial atomic velocities. The four distinguished orientations of fisetin inside the β-CD cavity obtained from docking were tested and compared to find the most preferential fisetin/β-CD inclusion complex. The ligand binding mode and water accessibility, host–guest interaction, and binding free energy of the inclusion complex were analyzed. The MM-PBSA/GBSA and M06-2X/6-31G(d,p)//MM-PBSA/GBSA approaches were used to predict the binding affinity of fisetin/β-CD complexes. The M06-2X/6-31G(d,p) optimization in gas phase and in water (Polarizable Continuum Model, PCM), also including BSSE correction was performed on this system.

## Materials and Methods

### System preparation

The two possible conformations of fisetin (CAS 528-48-3), resulting from a single bond rotation between the chromone and phenyl rings were optimized by the HF/6-31(d) method using Gaussian 03 program [34], while the β-CD structure was taken from our previous study [35]. To obtain the inclusion complex, each conformation of fisetin guest molecule was docked with 500 independent runs into the β-CD host cavity using the CDOCKER module in the Accelrys Discovery Studio Visualizer 3.0 program. Consequently, the docked complexes in solution were performed with the multiple MD simulations using the AMBER 10 software package [36]. The Glycam06 force field [37] was used to treat β-CD, while the atomic charges and parameters of fisetin were obtained from our previous study [38]. The hydrogen atoms of the host and guest molecules added by the LEaP module were minimized with 1000 steps of the steepest descents (SD) method followed by 2000 steps of the conjugated gradients (CG) method to release the bad contact. Afterwards, the inclusion complex was solvated by the SPC water molecules [39] with a spacing distance of 12 Å from the system surface. All systems consist of 1708 ± 48 water molecules in a 49.0 × 49.0 × 49.0 Å^3^ truncated octahedron periodic box. Then, the water molecules were only minimized with SD (2000 steps) and CG (1000 steps) continued by minimization of the whole system with the same minimized process for getting the initial structures to perform the MD simulations.

### Molecular dynamics simulations

Each MD simulation of fisetin/β-CD inclusion complexes was performed by the AMBER 10 software package coupled with the SANDER module in accordance with our previous studies [40–42]. The particle-mesh of Ewald’s method [43] was used for the treatment of the long-range electrostatic interactions with 12 Å cutoff distance. In order to constrain all bonds with hydrogen atoms, the SHAKE algorithm [44] was applied using a time step of 2 fs. The models were then heated up to 298 K with the relaxation time of 100 ps at constant volume up to 1 g/mL of water density. All systems were simulated using NPT ensemble at constant pressure of 1 atm equilibrated at 298 K for 70 ns. Temperature and pressure were controlled by the Berendsen weak coupling algorithm [45].

For analysis, the ptraj module of AMBER 10 program was used to evaluate the root mean square displacement (RMSD), the distance between the centers of gravity of each fisetin ring and β-CD, and the water accessibility to the ligand heteroatoms based on the radial distribution function (RDF). The calculations of MM-PBSA/GBSA binding free energies (∆*G*_MM-PBSA_ and ∆*G*_MM-GBSA_) and their energy components were analyzed using the mm_pbsa module.

### Free energy prediction

The MM-PBSA and MM-GBSA methods have been widely used to estimate the binding free energies of complex systems [46–50]. Herein, the binding free energy of the inclusion complex (Δ*G*_bind_) was calculated by the free energy difference of the complex (Δ*G*_complex_) and the isolated β-CD (Δ*G*_β-CD_) and fisetin (Δ*G*_fisetin_) molecules according to the following equation.

[1]
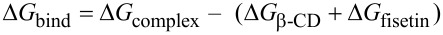


The total Gibbs free energy (Δ*G*) can be calculated from enthalpy (Δ*H*) and entropy terms with constant temperature (*T*Δ*S*).

[2]
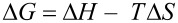


In solution, the Δ*H* term was divided into enthalpy energy in gas phase upon formation of complex (Δ*E*_MM_) and the free energy of solvation (Δ*G*_sol_), while the entropy term, *T∆S*, for conformational entropy change of the two individual molecules upon complexation process was taken from the normal mode analysis. Therefore, Equation 2 can be rewritten as:

[3]
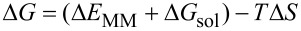


where Δ*E*_MM_ is the energy of molecular mechanics composed of bonded and non-bonded energies. The latter one contains the electrostatic (Δ*E*_ele_) and van der Waals interaction energies (Δ*E*_vdW_).

[4]
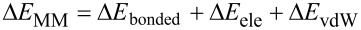


The solvation free energy term, Δ*G*_sol_, is comprised of polar and non-polar solvation terms. The polar solvation free energy term can be estimated from either the Poisson–Boltzmann (PB) or the generalized Born (GB) method.

[5]
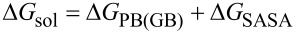


The nonpolar solvation free energy term, Δ*G*_SASA_, is estimated from a linear relation as:

[6]
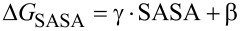


where SASA is the solvent-accessible surface area. The γ and β with the values of 0.00542 kcal/mol·Å^2^ and 0.92 kcal/mol, respectively, are taken from linear regression of a set of small nonpolar molecules solvent free energy in water [48,51–52].

In addition, the binding free energies were also corrected with quantum mechanics energy (∆*E*_QM_) by replacing the MM energy (∆*E*_MM_) in Equation 3 with density functional theory (DFT) calculation using the M06-2X functional with 6-31G(d,p) level of basis set.

Besides, the full optimization in gas phase and PCM water model of the representative inclusion complex was performed by using the M06-2X/6-31G(d,p) method. The BSSE correction was also taken into account.

## Results and Discussion

### Possible inclusion complexes

**Figure 3:**
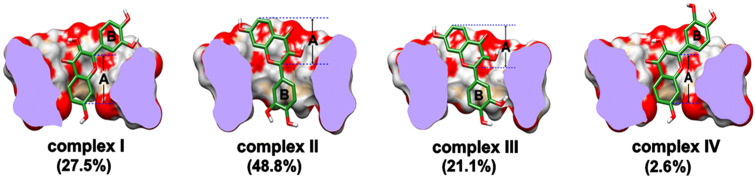
Docked structures of the four possible inclusion complexes between fisetin and β-CD, where their percentages of occurrence are given in parentheses.

Taking into account 1000 docked structures, two different groups of orientations of the fisetin guest molecule in the inclusion complex were observed (Figure 3). The chromone ring (A-ring) of fisetin was dipped into the hydrophobic cavity of β-CD, found in complexes **I** and **IV** (27.5 and 2.6% of occurrence, respectively). In contrast, the phenyl ring (B-ring) was occupied in the cavity instead for complexes **II** (48.8%) and **III** (21.1%). By considering the percentage of occurrence, it can be implied that complexation with β-CD was preferentially formed through the phenyl ring of fisetin. However, molecular docking in the gas phase may be insufficient for the determination of the structure and the stability of the inclusion complex in solution. To gain detailed insight in the energetic behavior and the geometry of the fisetin/β-CD complex of all four possible inclusion complexes (**I–IV**) in aqueous solution, MD simulations were then performed with three time repeats for each complex at different initial velocities, leading to altogether twelve simulated systems. Most stable structures with the highest amount of hydrogen bonding between fisetin and β-CD were chosen.

### System stability

**Figure 4:**
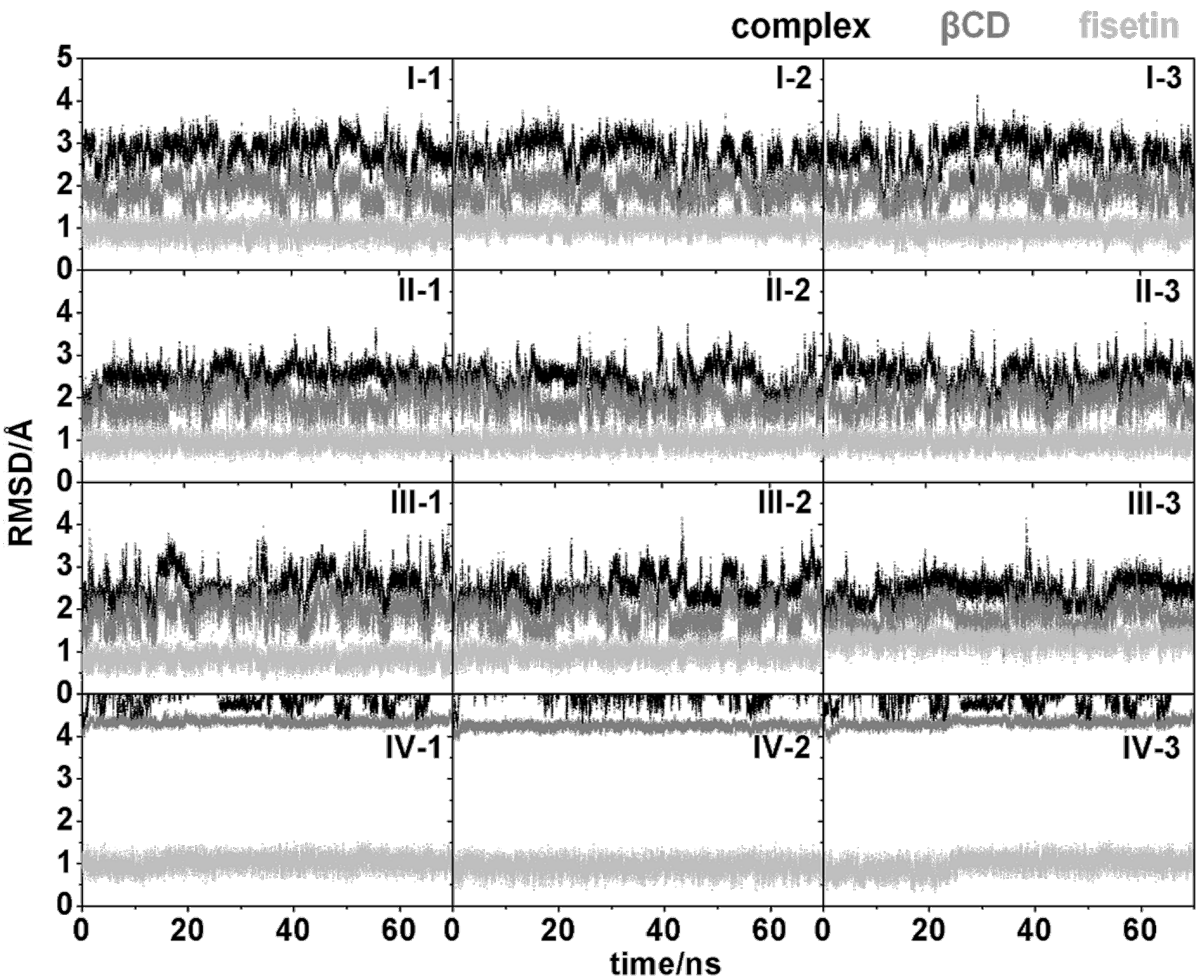
RMSD plots of all atoms in inclusion complex (black), β-CD (dark grey) and fisetin (light grey) for the twelve simulated systems of complexes **I–IV**.

To get some information about the system stability after equilibration of the inclusion complex, the root mean square displacement (RMSD) for all atoms of the complex, β-CD and fisetin relative to those of the initial structure from docking was calculated along the simulation time using the ptraj module of the AMBER 10 program. The RMSD plots for the twelve independent simulated systems are shown in Figure 4. In the complexes **I–III**, the RMSD values of fisetin (light gray) and β-CD (dark gray) were mostly found at ~1.0 and ~1.8 Å, respectively, consequently leading to rather stable inclusion complexes (RMSD values for **I**: ~2.8 Å and for **II–III**: ~2.5 Å). However, the complex **IV** was found to behave quite different from the other complexes. Its RMSD values of β-CD and complex increased up to ~4.3 and >5 Å, respectively, even though the other starting structures, taken randomly from docking results (12 structures from the total 26 structures), were selected. These simulations suggested that complex **IV** is likely unstable and may not occur in solution. Therefore, only the inclusion complexes **I–III** were further analyzed by using the MD trajectories from 10 to 70 ns.

### Fisetin binding mode

**Figure 5:**
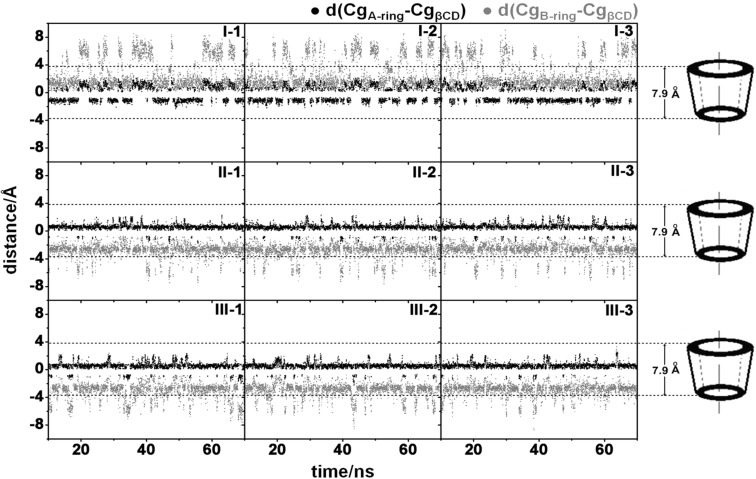
Distance between the centers of gravity of each fisetin ring (A/B) and β-CD along the simulation time for the three focused inclusion complexes **I-III**.

To understand the fisetin behavior inside the β-CD cavity along the simulation, the distance between the centers of gravity of each fisetin ring (Cg_ring_) and β-CD (Cg_β-CD_), d(Cg_ring_-Cg_β-CD_), was measured and plotted in Figure 5 for the last 60 ns simulation. If the Cg_β-CD_ is kept fixed as a reference point with orientation sketched in Figure 5 and the Cg_ring_ is calculated as the displacement, the negative and positive distance values are related to the position of the fisetin ring under and above Cg_β-CD_ in direction to the primary and secondary rims (approximately positioned at −3.95 and 3.95 Å on y-axis), respectively. The dashed line in Figure 5 represents the β-CD height of 7.9 Å [17].

For complex **I**, the A- and B-rings mainly located at ~−1.3 ± 0.6 and ~1.8 ± 1.1 Å suggesting that the fisetin was likely inserted into the hydrophobic cavity of β-CD. However, there was about 30% probability of fisetin translocation in which the B-ring passed through the wider rim of cyclodextrin, while the A-ring stayed above the CD center as seen by an increase in the d(Cg_B-ring_-Cg_β-CD_) to approximately 6.4 ± 1.2 Å and the d(Cg_A-ring_-Cg_β-CD_) to 1.1 ± 0.6 Å. The situation is different for the complexes **II** and **III**, where the B-ring binding is close to the primary rim instead. The small B-ring shows a better fit at the narrower rim of cyclodextrin (~−2.9 ± 0.9 Å) whereas the A-ring is located at the center of the cavity (~0.5 ± 0.4 Å). Only less than 10% occurrence of the B-ring moving through the primary rim (d(Cg_B-ring_ − Cg_β-CD_) < −4 Å) was observed. More frequent translocation was previously detected in the simulations of naringenin/β-CD complex due to the non-planarity and subsequently high flexibility of the guest molecule [42].

Interestingly, the simulations showed the translocation behavior of fisetin sometimes in the complex **I** and rarely in the complexes **II** and **III** instead of flip-flop movement because the fisetin molecule has never been moved out completely of the β-CD cavity. On the other hand, it could be implied that complexes **II** and **III** were more stable than complex **I**. It is worth to note that the three independent simulations for each complex gave rather conclusive evidence.

### Fisetin conformation

To monitor the conformational change and the flexibility of fisetin structure upon the three different formations of the inclusion complexes (**I–III**), the considered orientations between the chromone ring (A-ring) and phenyl ring (B-ring), defined as the O^1^–C^2^–C^1'–^C^2'^ torsional angle (τ, defined in Figure 1), were determined. The highest probability of torsional angles in the complexes **I**, **II** and **III** was found at 0 ± 50°, 10 ± 50° and −175 ± 30°, respectively. This suggested that no conformational change of fisetin structure occurred during the simulation, although the fisetin molecule is quite flexible (a large standard deviation value of 30–50°).

#### Solvation

**Figure 6:**
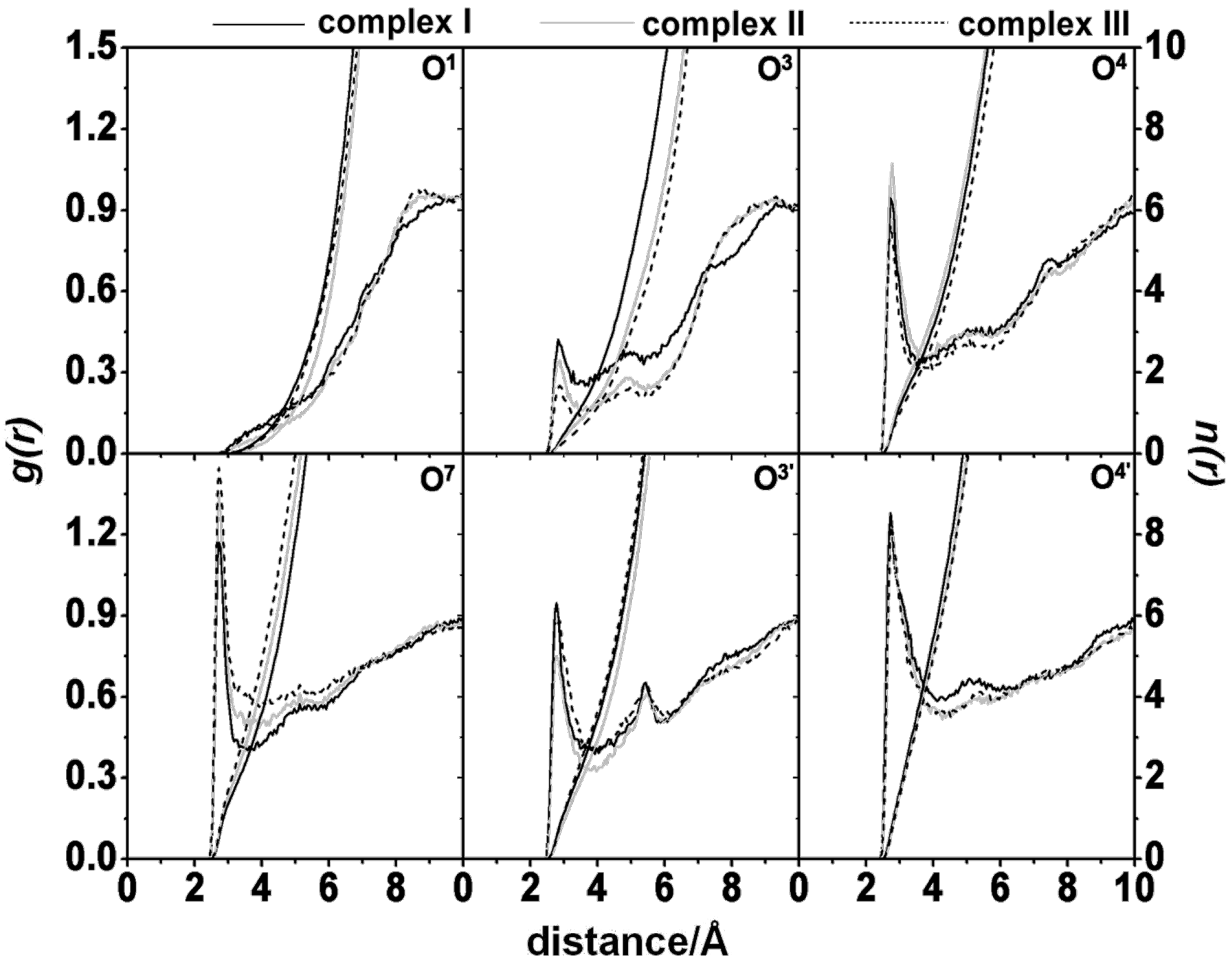
Radial distribution function (RDF) of oxygen atom of water molecules around the heteroatoms of fisetin in the complexation with β-CD for the three focused systems: complexes **I** (solid black line), **II** (solid grey line) and **III** (dashed line).

In this study, the radial distribution function (RDF, *g_ij_*(*r*)) calculation was used to monitor the water molecules (the oxygen atom of water *j*) in the spherical radius *r* of the fisetin heteroatom (oxygen atom *i*) in each complex. The RDF plots coupled with the integration number, *n*(*r*), averaged from the three independent simulations for each form of complexes **I–III** are shown in Figure 6 while the *n*(*r*) up to the first minimum is summarized in Table 1.

**Table 1:** Integration number, *n*(*r*), up to the first minimum from Figure 6 around the heteroatoms of fisetin in complexes **I–III**.

Atom	*n*(*r*)		
	complex **I**	complex **II**	complex **III**

O^1^ O^3^ O^4^ O^7^ O^3'^ O^4'^	– 1.5 2.2 2.5 3.5 5.7	– 0.8 2.3 4.0 3.0 6.0	– 0.6 2.1 4.5 3.1 6.0

The RDF plots give the information about the distribution of water oxygen atoms around all selected oxygen atoms of fisetin. From the atom–atom interaction RDF analysis, among all six oxygen atoms of fisetin in three complexes, no peak appeared within ~3 Å of the O^1^ atom, suggesting that this atom on the chromone ring has a relatively low water accessibility or very weak hydration interaction. Along the simulation this atom always seems to stay inside the hydrophobic cavity. Differentially, the other oxygen atoms show the first sharp peak centered at ~2.8 Å corresponding to a highly possible hydration and the first minimum at ~4 Å accounting for the time when a water molecule remains on the first solvation shell. On the opposite side of the O^1^ atom, the peak densities of the carbonyl oxygen O^4^ were almost identical with *n*(*r*) of ~2.2 for all complexes. A significant difference in the first shell of solvation for the two binding orientations of fisetin in the β-CD cavity (complex **I** and complexes **II–III**) was found for the other oxygen atoms. In complex **I**, more water molecules can be accessible to solvate the O^3^ and O^3'^ atoms located at the wider rim of cyclodextrin by 0.7/0.9 and 0.5/0.4 molecules relative to those of complex **II**/**III**, respectively. By the well-formed encapsulation of fisetin in the hydrophobic cavity of β-CD through the B-ring fitting at the narrow rim (complexes **II–III**), only the O^7^ atom on the A-ring was significantly higher solvated.

The further discussion on the hydration of fisetin in complexation with β-CD is summarized as follows. Low hydration on the O^3^ atom (**I–III**: ~1.5, ~0.8 and ~0.6) was found because it was mostly enclosed in the β-CD inner surface. In contrast, the exposed O^4'^ atom close to the secondary or primary rim in complex **I** or complexes **II–III** is much more solvated by water molecules (~6).

#### Binding free energy of inclusion complex

The MM-PBSA/GBSA approach is the energy calculation for estimating the binding free energies or calculating the free energies of molecules in solution. This method combines the molecular mechanical energies with the calculations of solvation. In order to calculate the electrostatic distribution to the free energy of solvation with a numerical solver, the Poisson–Boltzmann (PB) and generalized Born (GB) methods from the AMBER 10 program were applied. The 100 MD snapshots extracted from the production phase in each system were used for binding free energy calculations. The binding free energies (∆*G*) and the other energy contributions are given in Table 2, where the decomposition binding free energies from the A- and B-rings are shown in Table S1 of Supporting Information File 1.

**Table 2:** MM- and QM-PBSA/GBSA binding free energies (kcal/mol) and their energy components for the nine systems of the fisetin/β-CD complexes.

	Complex I			Complex II			Complex III		
	**I-1**	**I-2**	**I-3**	**II-1**	**II-2**	**II-3**	**III-1**	**III-2**	**III-3**

∆*E*_ele_	−8.6 ± 4.9	−9.2 ± 6.1	−8.6 ± 5.1	−9.4 ± 4.6	−9.1 ± 3.8	−9.7 ± 4.1	−9.3 ± 5.3	−10.9 ± 5.4	−9.8 ± 4.6
∆*E*_vdW_	−28.6 ± 3.3	−28.8 ± 3.2	−28.9 ± 2.8	−30.8 ± 3.1	−31.3 ± 2.6	−31.1 ± 2.8	−30.1 ± 2.6	−29.7 ± 2.5	−30.3 ± 3.0
∆*E*_MM_	−37.2 ± 5.4	−38.0 ± 5.7	−37.5 ± 5.3	−40.2 ± 5.5	−40.3 ± 4.3	−40.8 ± 4.0	−39.5 ± 5.5	−40.6 ± 5.9	−40.1 ± 5.5
∆*E*_QM_	−28.4 ± 5.1	−29.3 ± 5.1	−28.6 ± 4.9	−31.0 ± 5.5	−31.7 ± 4.8	−32.4 ± 4.6	−32.0 ± 5.8	−33.4 ± 6.4	−32.0 ± 6.2
*T*∆*S*	−17.2 ± 3.1	−17.0 ± 4.8	−17.1 ± 2.7	−16.9 ± 3.2	−16.6 ± 3.2	−17.3 ± 2.7	−16.2 ± 2.5	−17.3 ± 3.5	−16.8 ± 3.3
∆*G*_sol(PBSA)_	9.3 ± 2.3	9.7 ± 2.5	9.3 ± 2.1	10.3 ± 2.0	10.2 ± 1.9	10.3 ± 1.9	−10.3 ± 2.6	11.1 ± 2.7	10.8 ± 2.3
∆*G*_sol(GBSA)_	9.0 ± 2.2	9.3 ± 2.6	9.2± 2.5	9.6 ± 2.0	9.5 ± 1.9	9.7 ± 1.7	9.3 ± 2.4	10.0 ± 2.4	9.7 ± 2.1
∆*G*_MM-PBSA_	−10.7 ± 1.9	−11.2 ± 2.1	−11.1 ± 1.8	−13.0 ± 1.9	−13.5 ± 1.8	−13.2 ± 1.7	−13.0 ± 1.8	−12.2 ± 1.9	−12.5 ± 1.9
∆*G*_MM-GBSA_	−11.0 ± 1.9	−11.7 ± 2.1	−11.2 ± 1.9	−13.7 ± 1.9	−14.2 ± 1.8	−13.8 ± 1.7	−14.0 ± 1.9	−13.3 ± 2.0	−13.6 ± 1.9
∆*G*_QM-PBSA_	−1.9 ± 2.1	−2.6 ± 1.7	−2.2 ± 2.1	−3.8 ± 2.1	−4.9 ± 1.9	−4.8 ± 1.9	−5.5 ± 2.4	−5.0 ± 2.4	−4.4 ± 2.3
∆*G*_QM-GBSA_	−2.2 ± 2.0	−3.0 ± 1.7	−2.3 ± 2.2	−4.5 ± 2.1	−5.6 ± 1.9	−5.4 ± 1.9	−6.5 ± 2.4	−6.1 ± 2.3	−5.5 ± 2.2

**Figure 7:**
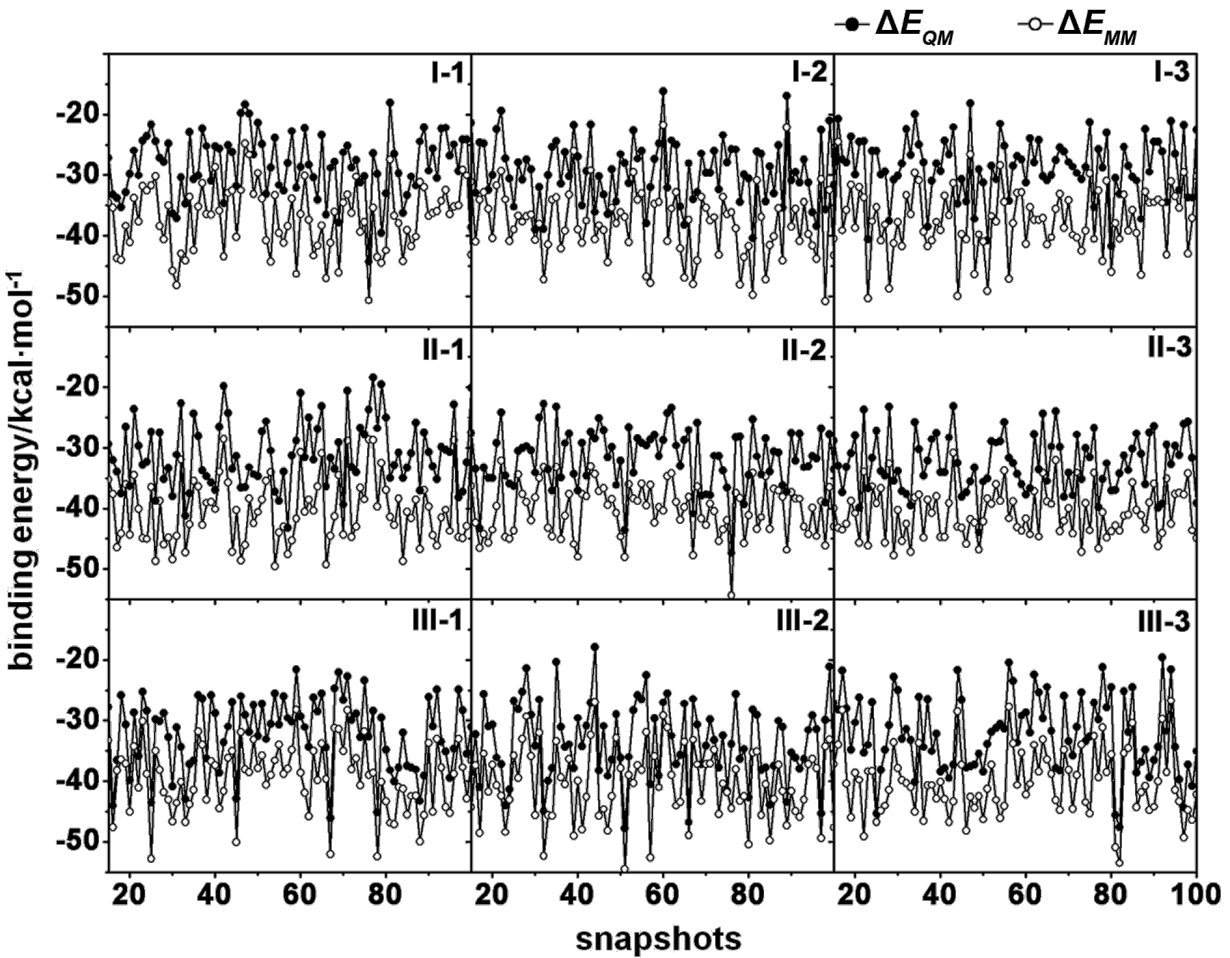
Comparison between QM and MM energies (∆*E*_QM_ and ∆*E*_MM_) per the same set of 100 MD snapshots in the three formed inclusion complexes **I–III**.

By molecular mechanics (MM) calculation in gas phase, the attractive electrostatic contributions (∆*E*_ele_) between fisetin and β-CD were similar in all three complexes (−9 to −11 kcal/mol), whilst slightly stronger van der Waals interactions (∆*E*_vdW_) by 1–2 kcal/mol were observed in the complexes **II** and **III**. As expected, the vdW force is the key interaction in forming the inclusion complex as seen by approximate 3-fold stronger interaction than electrostatic energy. Based on ∆*E*_MM_ calculated values, the complexes **II** and **III** (~ −40 kcal/mol) were ~3 kcal/mol more stable than complex **I**. In Table S1 of Supporting Information File 1, the enhanced stability of these two complexes was mainly contributed from the B-ring by 2 kcal/mol, while the A-ring almost equally stabilized either complex **II–III** or complex **I**. Since the binding interaction may not be accurately described by the MM method, the DFT single point calculations with M06-2X/6-31G(d,p) level of theory were applied on the same set of MD snapshots for each system. On the basis of all calculations with a summation of solvation free energy, either MM-PBSA/GBSA or QM-PBSA/GBSA established the same conclusive evidence of a better formation of inclusion complexes **II** and **III** than complex **I**. The QM-PBSA/GBSA methods were able to predict the Gibbs free energy of the fisetin/β-CD complex comparatively close to the experimental value of ~−4 kcal/mol [28,33]. The preferable formation of inclusion complex through the B-ring (complex **II**) was previously proposed from the reaction path study with SAM1 semi-empirical method [28]; however, the calculated ∆*G* of such complex was in the range of 1.6–4.4 kcal/mol. Additionally, it is worth to note that in comparison to the QM energy (∆*E*_QM_) the MM method was likely found to overestimate the binding interaction by ca. 10 kcal/mol for all snapshots in the three forms of complex (Figure 7). Our results also suggested that for binding free energy prediction, the entropy and solvation terms were important factors.

In addition, the complexation energy of fisetin binding to β-CD (complex **I** selected as the representative system) was performed at the M06-2X/6-31G(d,p) level of theory. Its binding energies in gas phase and PCM water were of −38.9 and −30.7 kcal/mol, respectively. With BSSE correction, the complexation energies of the investigated system become −16.6 and −8.9 kcal/mol somewhat closer to the experimental data. The results suggested that neither solvation effect nor basis set superposition error can be neglected.

#### Intermolecular hydrogen bond

In order to monitor intermolecular hydrogen bonds of inclusion complex during the simulation time, hydrogen bonds between the fisetin and β-CD can be evaluated in terms of the percentage of hydrogen bond occupation in accordance with the geometric criteria of (i) a distance between the proton donor (D) and acceptor (A) atoms ≤3.5 Å and (ii) the D–H···A angle of ≥120°. The results are presented in Table 3. As expected, the rather low hydrogen bond occupations between fisetin and β-CD of <40% were observed in consistence with an important role of vdW force in inclusion complex as mentioned previously. From Table 3, there were five possible hydrogen bonds in complex I and only three hydrogen in the other two complexes, where the pair of hydrogen bond formation and interaction strength likely depended on the orientation of fisetin inside the cavity of β-CD. In all complexes, the carbonyl oxygen (O^4^) on the A-ring weakly interacted with the O^2^ and O^3^ atoms at the secondary rim of β-CD. In complex I, the O^3'^ atom on B-ring was able to make weak hydrogen bonds with both oxygen atoms O^2^ and O^3^ at the wider rim, but in complexes **II** and **III** they slightly interacted with the O^6^ at the narrower rim instead. In addition, a weak hydrogen bond between the O^7^ atom on A-ring and the O^6^ of β-CD was only detected in complex **I**.

**Table 3:** Percentage of hydrogen bond occupations for the nine systems of the fisetin/β-CD complexes.

hydrogen bonding interactions	% hydrogen bond occupation								

	Complex **I**			Complex **II**			Complex **III**		
	**I-1**	**I-2**	**I-3**	**II-1**	**II-2**	**II-3**	**III-1**	**III-2**	**III-3**

O^4^(A-ring)···H–O^2^	4	5	3	13	11	10	23	25	23
O^7^(A-ring)···H–O^6^	13	11	12	–	–	–	–	–	–
O^3'^(B-ring)···H–O^2^	37	31	37	–	–	–	–	–	–
O^3'^(B-ring)···H–O^3^	26	25	24	–	–	–	–	–	–
O^3'^(B-ring)···H–O^6^	–	–	–	23	28	24	11	14	15
O^3^–H(A-ring)···O^4^	7	9	9	3	3	3	9	9	9

## Conclusion

In this study, multi-MD simulations were applied to investigate the complexation of fisetin with β-CD in aqueous solution. Molecular docking suggested that there are four possible binding modes of fisetin in complex with β-CD at a 1:1 ratio. For complexes **I** and **IV**, the chromone ring of fisetin occupied the hydrophobic cavity, but the phenyl ring was encapsulated in complexes **II** and **III**. The 3'-OH group on the phenyl ring was positioned on the same side of the O^1^ atom as for complexes **I** and **IV** and in vice versa for the other two complexes. By the multiple simulations on the twelve different starting structures, the complex **IV** seems to be unfavorable in aqueous solution. The translocation of the fisetin molecule inside the β-CD cavity was more likely observed in complex **I** than in complexes **II** and **III**. Although the distinct patterns of water accessibility towards the fisetin oxygen atoms were found in different binding modes, the atoms exposed on the wider rim of cyclodextrin are reasonably higher solvated, except for the O^4'^ atom with the highest solvation in all complexes. MM- and QM-PBSA/GBSA approaches suggested that the phenyl ring of fisetin was more preferable to reside in the β-CD cavity forming a stable complex. The predicted binding free energies based on QM-PBSA/GBSA methods were comparable with the experimental data. The MM energy components indicated that van der Waals interaction is the main force in forming the inclusion complex. From the QM calculation at the M06-2X/6-31G(d,p) level of theory, both, the solvation effect and the BSSE correction were found to be factors in predicting the complexation energy of the considered system.

## Supporting Information

File 1 Decomposition of the free energy (kcal/mol) into the contributions from A- and B-rings of fisetin.

## References

[1] Cook N C, Samman S (1996). J Nutr Biochem.

[2] Havsteen B H (2002). Pharmacol Ther.

[3] Havsteen B (1980). Z Lebensm-Unters Forsch.

[4] Narayana K R, Reddy M S, Chaluvadi M R, Krishna D R (2001). Indian J Pharmacol.

[5] Constantin R P, Constantin J, Pagadigorria C L S, Ishii-Iwamoto E L, Bracht A, Kássia Cardoso Ono M, Yamamoto N S (2010). Cell Biochem Funct.

[6] Kimira M, Arai Y, Shimoi K, Watanabe S (1998). J Epidemiol.

[7] Nijveldt R J, van Nood E, van Hoorn D E, Boelens P G, van Norren K, van Leeuwen P A (2001). Am J Clin Nutr.

[8] Ishige K, Schubert D, Sagara Y (2001). Free Radical Biol Med.

[9] Sagara Y, Vanhnasy J, Maher P (2004). J Neurochem.

[10] Maher P, Akaishi T, Abe K (2006). Proc Natl Acad Sci U S A.

[11] Chen Y-C, Shen S-C, Lee W-R, Lin H-Y, Ko C-H, Shih C-M, Yang L-L (2002). Arch Toxicol.

[12] Lee W-R, Shen S-C, Lin H-Y, Hou W-C, Yang L-L, Chen Y-C (2002). Biochem Pharmacol.

[13] Suh Y, Afaq F, Johnson J J, Mukhtar H (2009). Carcinogenesis.

[14] Ravichandran N, Suresh G, Ramesh B, Vijaiyan Siva G (2011). Food Chem Toxicol.

[15] Hanneken A, Lin F-F, Johnson J, Maher P (2006). Invest Ophthalmol Visual Sci.

[16] Ragelle H, Crauste-Manciet S, Seguin J, Brossard D, Scherman D, Arnaud P, Chabot G G (2012). Int J Pharm.

[17] Szejtli J (1998). Chem Rev.

[18] Loftsson T, Brewster M E (1996). J Pharm Sci.

[19] Boonyarattanakalin K, Wolschann P, Toochinda P, Lawtrakul L (2012). Eur J Pharm Sci.

[20] Rusa C C, Luca C, Tonelli A E (2001). Macromolecules.

[21] Banerjee A, Sengupta B, Chaudhuri S, Basu K, Sengupta P K (2006). J Mol Struct.

[22] Murphy R S, Barros T C, Mayer B, Marconi G, Bohne C (2000). Langmuir.

[23] Chen W, Chang C-E, Gilson M K (2004). Biophys J.

[24] Chittiteeranon P, Soontaros S, Pongsawasdi P (2007). J Inclusion Phenom Macrocyclic Chem.

[25] Saikosin R, Limpaseni T, Pongsawasdi P (2002). J Inclusion Phenom Macrocyclic Chem.

[26] Lipkowitz K B (1998). Chem Rev.

[27] Reddy M N, Rehana T, Ramakrishna S, Chowdary K P, Diwan P V (2004). AAPS PharmSci.

[28] Guzzo M R, Uemi M, Donate P M, Nikolaou S, Machado A E H, Okano L T (2006). J Phys Chem A.

[29] Zhang H, Feng W, Li C, Tan T (2010). J Phys Chem B.

[30] Zheng Y, Chow A H L, Haworth I S (2008). Lett Drug Des Discovery.

[31] Choi Y-J, Lee J-H, Cho K-W, Hwang S-T, Jeong K-J, Jung S-H (2005). Bull Korean Chem Soc.

[32] Chakraborty S, Basu S, Lahiri A, Basak S (2010). J Mol Struct.

[33] Banerjee A, Sengupta P K (2006). Chem Phys Lett.

[34] (2004). Gaussian 03.

[35] Snor W, Liedl E, Weiss-Greiler P, Karpfen A, Viernstein H, Wolschann P (2007). Chem Phys Lett.

[36] (2008). AMBER 10.

[37] Kirschner K N, Yongye A B, Tschampel S M, González-Outeiriño J, Daniels C R, Foley B L, Woods R J (2008). J Comput Chem.

[38] Khuntawee W, Rungrotmongkol T, Hannongbua S (2012). J Chem Inf Model.

[39] Jorgensen W L, Chandrasekhar J, Madura J D, Impey R W, Klein M L (1983). J Chem Phys.

[40] Rungrotmongkol T, Arsawang U, Iamsamai C, Vongachariya A, Dubas S T, Ruktanonchai U, Soottitantawat A, Hannongbua S (2011). Chem Phys Lett.

[41] Rungnim C, Arsawang U, Rungrotmongkol T, Hannongbua S (2012). Chem Phys Lett.

[42] Sangpheak W, Khuntawee W, Wolschann P, Pongsawasdi P, Rungrotmongkol T (2014). J Mol Graphics Modell.

[43] York D M, Darden T A, Pedersen L G (1993). J Chem Phys.

[44] Ryckaert J-P, Ciccotti G, Berendsen H J C (1977). J Comput Phys.

[45] Berendsen H J C, Postma J P M, van Gunsteren W F, DiNola A, Haak J R (1984). J Chem Phys.

[46] Decha P, Rungrotmongkol T, Intharathep P, Malaisree M, Aruksakunwong O, Laohpongspaisan C, Parasuk V, Sompornpisut P, Pianwanit S, Kokpol S (2008). Biophys J.

[47] Rungrotmongkol T, Nunthaboot N, Malaisree M, Kaiyawet N, Yotmanee P, Meeprasert A, Hannongbua S (2010). J Mol Graphics Modell.

[48] Kaiyawet N, Rungrotmongkol T, Hannongbua S (2013). J Chem Inf Model.

[49] Rastelli G, Del Rio A, Degliesposti G, Sgobba M (2010). J Comput Chem.

[50] Beà I, Gotsev M G, Ivanov P M, Jaime C, Kollman P A (2006). J Org Chem.

[51] Sitkoff D, Sharp K A, Honig B (1994). J Phys Chem.

[52] Genheden S, Kongsted J, Söderhjelm P, Ryde U (2010). J Chem Theory Comput.

