# Supplementary material for: Binding mode and free energy prediction of fisetin/β-cyclodextrin inclusion complexes
Source: Beilstein J Org Chem. 2014 Nov 27;10:2789–99. doi: 10.3762/bjoc.10.296 (PMC4273227; doi:10.3762/bjoc.10.296)
Supplement: File 1 — Decomposition of the free energy (kcal/mol) into the contributions from A- and B-rings of fisetin. [file Beilstein_J_Org_Chem-10-2789-s001.pdf]

Supporting Information

for

Binding mode and free energy prediction of fisetin/ β-cyclodextrin inclusion complexes

Bodee Nutho[1], Wasinee Khuntawee[2], Chompoonut Rungnim[3], Piamsook Pongsawasdi[1], Peter Wolschann[4,5], Alfred Karpfen[5], Nawee Kungwan[6], Thanyada Rungrotmongkol*[,§,1]

Address: [1]Department of Biochemistry, Faculty of Science, Chulalongkorn University, Bangkok 10330, Thailand, [2]Nanoscience and Technology Program, Graduate School, Chulalongkorn University, Bangkok, 10330, Thailand, [3]National Nanotechnology Center (NANOTEC), National Science and Technology Development Agency (NSTDA), 111 Thailand Science Park, Thanon Phahonyothin Tambon Khlong Nueng, Amphoe Khlong Luang, Pathum Thani 12120, Thailand, [4]Department of Pharmaceutical Technology and Biopharmaceutics, University of Vienna, Vienna 1090, Austria, [5]Institute of Theoretical Chemistry, University of Vienna, Vienna 1090, Austria and [6]Department of Chemistry, Faculty of Science, Chiang Mai University, Chiang Mai 50200, Thailand

Email: Thanyada Rungrotmongkol* t.rungrotmongkol@gmail.com

[*]Corresponding author.

[§]Tel: +66 2 2185426; Fax: + 66 22 185418.

Decomposition of the free energy (kcal/mol) into the contributions from A- and B-rings of fisetin

Table S1: Decomposition of the free energy (kcal/mol) into the contributions from A- and B-rings of fisetin for the nine systems of the fisetin/βCD complexes

	$\Delta G_{MM\text{-}PBSA}$			$\Delta G_{MM\text{-}GBSA}$		
	Total	A-ring	B-ring	Total	A-ring	B-ring
**Complex I**						
I-1	-27.9±3.8	-16.0±4.1	-11.9±4.4	-23.8±3.1	-14.4±3.7	-9.4±4.3
I-2	-28.2±3.8	-16.4±3.8	-11.8±3.8	-23.9±2.8	-14.7±3.5	-9.2±4.2
I-3	-28.2±3.7	-15.8±4.1	-12.4±4.5	-24.1±2.5	-14.3±3.4	-9.8±4.2
**Complex II**						
II-1	-29.9±4.3	-15.6±3.5	-14.3±2.8	-26.1±2.9	-15.0±2.6	-11.1±2.3
II-2	-30.1±3.1	-16.5±3.0	-13.6±3.0	-26.4±2.3	-15.6±2.4	-10.8±2.4
II-3	-30.5±3.1	-16.4±3.0	-14.1±2.8	-26.2±2.4	-15.3±2.4	-10.9±2.3
**Complex III**						
III-1	-29.2±3.7	-16.1±3.5	-13.1±3.3	-25.5±2.5	-15.0±2.4	-10.5±2.3
III-2	-29.5±3.7	-15.7±3.6	-13.8±3.6	-25.4±2.3	-14.8±2.4	-10.6±2.4
III-3	-29.3±3.9	-15.2±3.5	-14.1±3.1	-25.5±2.9	-14.7±2.4	-10.8±2.0
